# Knowledge, Attitudes, and Practices of Breast Cancer Screening Methods Among Female Patients in Primary Healthcare Centers in Najran, Saudi Arabia

**DOI:** 10.1007/s13187-018-1423-8

**Published:** 2018-09-06

**Authors:** Majed Alshahrani, Sultan Yahya M. Alhammam, Hussain Ali Salem Al Munyif, Amani Mohammad Abbad Alwadei, Alanood Mohammad Abbad Alwadei, Soha Saleh Mohammed Alzamanan, Norah Saad M. Aljohani

**Affiliations:** 1 Department of Obstetrics and Gynecology, College of Medicine, Najran University, Najran, Saudi Arabia; 2 Najran University, Najran, Saudi Arabia; 3 Najran General Hospital, Najran, Saudi Arabia

**Keywords:** Breast cancer knowledge, Mammogram, Breast clinical examination, Self-examination, Breast cancer screening

## Abstract

The aim of the present study was to evaluate the knowledge, attitude, and practice of breast cancer screening methods among female patients attending five primary healthcare centers in Najran, Saudi Arabia. A cross-sectional survey was conducted on 500 female patients, and a direct interview questionnaire was used to collect data. Our results indicated a low knowledge about breast cancer, breast self-examination, mammogram and clinical breast examination was 54.4%, 56%, 90.4%, and 83.8%, respectively. Nineteen percent of patients demonstrated a high knowledge of breast self-examination. Breast self-examination was performed by 35% of patients, whereas 15% of patients received mammograms and 19.8% clinical breast examinations. The most common barrier for screening methods of breast cancer was that patients were unaware of half of the screening methods. A total of 20.6% of women did not perform breast self-examinations because they were not well trained, and 26.4% of women did not receive clinical breast examinations because there was no female doctor available. The main source of information among the women patients was social media (52.4%), whereas 8.8% received information through their healthcare provider. In summary, the majority of women demonstrated poor knowledge about breast cancer and screening methods. Additional effort should be put forth through women’s healthcare providers to increase the awareness of breast cancer screening. That means we need to continue emphasizing the importance of primary healthcare for early detection of breast cancer in the early stages.

**Electronic supplementary material:**

The online version of this article (10.1007/s13187-018-1423-8) contains supplementary material, which is available to authorized users.

## Introduction

Breast cancer is the second most common cancer worldwide, and most frequent among females [1]. The number of new cases diagnosed in 2012 was approximately 1.67 million (25% of all cancer cases) worldwide [1]. Breast cancer is the fifth cause of death from cancer (overall about 522.000 cases) [1]. Early detection of breast cancer and prompt treatment offer the greatest chance of long-term survival. Mammography, clinical breast examination, and breast self-examination are secondary prevention methods used for screening breast cancer [2].

In Saudi Arabia, breast cancer usually presents at advanced stages and more frequently in young pre-menopausal age women compared with Western countries [3]. According to the Ministry of Health in Saudi Arabia, the number of new cases of cancer is 2741 including about 19% of breast cancer in women that is ranked first figures [4]. In the USA, 50% of new breast cancer occurs in women over 65 years of age, whereas in Saudi Arabia, breast cancer usually occurs at the age of 52 years old. In addition, developed countries discover breast cancer mostly in the early stages compared with developing countries where there are large numbers diagnosed at advanced stages [4].

The Najran region is located in the south west corner of the Kingdom of Saudi Arabia. It is bordered in the north by Riyadh and Eastern Regions, in the east by the Eastern Region, in the south by Yemen, and in the west by the Aseer Region. The population census reported that Najran city has a population of 331,683 [5].

Many studies were conducted in Saudi Arabia regarding knowledge and practice of breast cancer screening methods. In a study conducted, the Breast Cancer Screening in Saudi Arabia: Free but Almost No Takers, it was found that among 10,735 participants of study 89% of women reported not having a clinical breast exam and 92% reported never having a mammogram [6]. Other studies conducted the patterns, knowledge, and barriers of mammography use among women in Saudi Arabia, and 3245 women were surveyed, with 40% reporting ever having a mammogram [7].

The awareness of breast cancer plays an important role in early detection and prevention of the disease, and the primary healthcare center is the first basic level of contact between women and families within the health system. Therefore, our study aimed to evaluate the knowledge, attitude, and practice of breast cancer screening methods among female patients attending primary healthcare centers.

## Materials and Methods

Our study consisted of a cross-sectional, interviewer-administered, questionnaire-based survey method. The study took place at five primary healthcare centers in Najran City, Saudi Arabia. All female patients who attended and registered at the five primary healthcare centers in Najran city who agreed to participate in the study were included with 100% response rate; no exclusion criteria was performed. The medical condition of participants was stable either antenatal care visit for pregnant women or follow-up of chronic medical disease like diabetes mellitus or hypertension. The study was conducted from September 1, 2017 to December 31, 2017. A total of 500 participants were included, with a margin of error at 5% and a confidence interval of 95%. An ethical approval letter was obtained from the research committee at the College of Medicine, Najran University, before the start of the study.

The researcher used a self-administered questionnaire consisting of the following: (1) sociodemographic data, including age, educational level, nationality, medical, and gynecology history; (2) knowledge of breast cancer, mammograms, clinical breast examinations, and breast self-examinations; (3) attitude, practice, and barrier for breast cancer screening; (4) source of information. The pilot study was conducted in 30 patients to evaluate the content of the questionnaire and the average time it took to complete. Then, corrections were made accordingly. These 30 pilot study cases were not included in the main study. Data was obtained by the research team who gave the questionnaire directly to the female patients. Each participant took 5 to 7 min to complete the questionnaire.

### Statistical Analysis

Data were collected, revised, coded, and entered into a statistical software program (IBM SPSS version 21). All statistical analysis was performed using two-tailed tests, with an alpha error of 0.05. *P* value less than or equal to 0.05 was considered to be statistically significant. Any correct answer was given a score of one point; otherwise, a score of zero was given. The discrete scores for the different knowledge domains were summed. Knowledge score was categorized into poor score for those who had a score of less than 50% of the maximum score, moderate knowledge if score was 50 to 75%, and good knowledge if score was above 75% of the maximum score. Descriptive statistics, including frequencies and percent, were used to describe the frequency of each response for the categorical data. Chi-square test/Monte Carlo exact test and Fisher’s exact test were used to test for associations between sample characteristics and knowledge level.

## Results

Five hundred (100%) women that participated in the study completed the interview questionnaire. The demographic and medical characteristics of the participants are shown in Online Resource 1. Approximately half of the participants demonstrated a low level of breast cancer knowledge (272/500 women, 54.4%) and breast self-examinations (284/500 women, 56.8%). More than 80% of women displayed a low level of mammogram knowledge (452/500 women, 90.4%) and knowledge related to clinical breast examinations (419/500 women, 83.8%). A total of 19% of participants had a high level of knowledge for breast self-examinations, 10.2% for general knowledge of breast cancer, 1.6% for mammogram, and 4.8% for clinical breast examinations.

We calculated the distributions of predictors for the general knowledge of breast cancer (Table 1). The highest predictors of general breast cancer knowledge were occupation (working vs. housewife), medical history (free vs. positive), and age of menarche (< 12 years old) (*P* = 0.001). The second predictor was education level (*P* = 0.006). In addition, women’s history of benign breast disease was a high predictor of breast cancer knowledge (*P* = 0.045).

**Table 1:** Distribution of the general knowledge of breast cancer in female patients attending primary healthcare centers in Najran City, Saudi Arabia

Predictor		General knowledge level						*P*
		Low		Moderate		High		
		No.	%	No.	%	No.	%	
Age in years	<30	90	57.0%	55	34.8	13	8.2	0.906
	30–39	115	53.2	79	36.6	22	10.2	
	40–49	53	53.0	35	35.0	12	12.0	
	50+	14	53.8	8	30.8	4	15.4	
Education level	Illiterate	40	61.5	20	30.8	5	7.7	0.006*
	Primary	48	61.5	19	24.4	11	14.1	
	Secondary	95	61.7	48	31.2	11	7.1	
	University	68	46.6	60	41.1	18	12.3	
	Postgraduate	21	36.8	30	52.6	6	10.5	
Occupation	Working	39	36.4	51	47.7	17	15.9	0.001*
	Housewife	233	59.3	126	32.1	34	8.7	
Marital status	Single	37	63.8	16	27.6	5	8.6	0.522
	Married	186	53.0	131	37.3	34	9.7	
	Divorced	33	58.9	16	28.6	7	12.5	
	Widow	16	45.7	14	40.0	5	14.3	
Medical history	Free	215	59.9	119	33.1	25	7.0	0.001*
	Positive	57	40.4	58	41.1	26	18.4	
History of benign breast disease	Yes	23	65.7	6	17.1	6	17.1	0.045*
	No	249	53.5	171	36.8	45	9.7	
Age of marriage	< 20	102	56.7	55	30.6	23	12.8	0.246
	20–29	107	50.7	88	41.7	16	7.6	
	30–39	24	50.0	17	35.4	7	14.6	
	40+	2	66.7	1	33.3	0	0.0	
Parity	Nulliparous	40	51.3	25	32.1	13	16.7	0.351
	Less than 4	134	54.0	93	37.5	21	8.5	
	4+	61	52.6	43	37.1	12	10.3	
Age of menarche	Unknown	57	51.8	34	30.9	19	17.3	0.001*
	< 12 years	105	66.5	38	24.1	15	9.5	
	12–14	87	45.5	92	48.2	12	6.3	
	After 14 years	23	56.1	13	31.7	5	12.2	
Age of menopause	Still menstruating	243	54.4	161	36.0	43	9.6	0.229
	Before 50 years	12	60.0	4	20.0	4	20.0	
	After 50 years	4	36.4	4	36.4	3	27.3	
	Don’t know	13	59.1	8	36.4	1	4.5	

*P < 0.05 (significant)

The distributions of predictors of breast cancer screening methods are shown in Table 2. The highest predictors of knowledge of breast cancer screening methods were education level, occupation, history of benign breast disease, and parity (*P* = 0.001). The age of marriage (*P* = 0.003), age of menarche (*P* = 0.005), and age of participants (*P* = 0.012) was a predictor of breast cancer screening knowledge.

**Table 2:** Distributions of cancer breast screening knowledge of female patients attending primary healthcare centers in Najran, Saudi Arabia

Predictor		Screening total knowledge level						*P*
		Low		Moderate		High		
		No.	%	No.	%	No.	%	
Age in years	< 30	130	82.3	19	12.0	9	5.7	0.012*
	30-39	179	82.9	35	16.2	2	.9	
	40–49	89	89.0	11	11.0	0	0.0	
	50+	24	92.3	2	7.7	0	0.0	
Education level	Illiterate	64	98.5	1	1.5	0	0.0	0.001*
	Primary	72	92.3	6	7.7	0	0.0	
	Secondary	136	88.3	17	11.0	1	.6	
	University	108	74.0	28	19.2	10	6.8	
	Postgraduate	42	73.7	15	26.3	0	0.0	
Occupation	Working	74	69.2	22	20.6	11	10.3	0.001*
	Housewife	348	88.5	45	11.5	0	0.0	
Marital status	Single	51	87.9	7	12.1	0	0.0	0.270
	Married	292	83.2	49	14.0	10	2.8	
	Divorced	45	80.4	10	17.9	1	1.8	
	Widow	34	97.1	1	2.9	0	0.0	
Medical history	Free	304	84.7	45	12.5	10	2.8	0.258
	Positive	118	83.7	22	15.6	1	.7	
History of benign breast disease	Yes	20	57.1	6	17.1	9	25.7	0.001*
	No	402	86.5	61	13.1	2	.4	
Age of marriage	< 20	164	91.1	16	8.9	0	0.0	0.003*
	20-29	166	78.7	34	16.1	11	5.2	
	30–39	39	81.3	9	18.8	0	0.0	
	40+	2	66.7	1	33.3	0	0.0	
Parity	Nulliparous	59	75.6	11	14.1	8	10.3	0.001*
	Less than 4	204	82.3	41	16.5	3	1.2	
	4+	108	93.1	8	6.9	0	0.0	
Age of menarche	Unknown	102	92.7	7	6.4	1	.9	0.005*
	< 12 years	132	83.5	18	11.4	8	5.1	
	12–14	155	81.2	34	17.8	2	1.0	
	After 14 years	33	80.5	8	19.5	0	0.0	
Age of menopause	Still menstruating	373	83.4	63	14.1	11	2.5	0.591
	Before 50 years	19	95.0	1	5.0	0	0.0	
	After 50 years	9	81.8	2	18.2	0	0.0	
	Don’t know	21	95.5	1	4.5	0	0.0	

*P < 0.05 (significant)

A total of 35% of women that attended the primary healthcare center performed breast self-examinations; 15% underwent a mammogram. Moreover, 19.8% of women visited their physician for a clinical breast examination. The remaining 30.2% have not had a breast screening method.

The barriers to breast cancer screening are shown in Table 3. A total of 57% of women participants were unaware of mammograms and 13.6% were afraid of the result. A low percentage of women (26.4%) have had clinical breast examinations because no female doctor was available and 22.2% were afraid of the result. Only 20.6% of women have performed breast self-examinations because of lack of training and 17.6% were afraid of the result.

**Table 3:** Barriers to breast screening recorded among female patients attending primary healthcare centers in Najran City, Saudi Arabia

Barriers		No.	%
Mammogram	No idea	285	57.0
	Harmful	57	11.4
	No facility	43	8.6
	Painful	47	9.4
	Free of results	68	13.6
Clinical breast examination	No idea	221	44.2
	No female doctors	132	26.4
	Painful	36	7.2
	Free of results	111	22.2
Breast self-examination	No idea	288	57.6
	No training	103	20.6
	Free of results	89	17.8
	Painful	20	4.0

More than half of women (52.4%) received information about breast cancer and screening methods from social media, whereas 8.8% obtained information from their healthcare providers. A total of 19.8% of women received information from television through health programs, 6.2% from magazines/newspapers, and 13.0% from other sources.

## Discussion

Breast cancer is the most common cancer in Saudi Arabia. Over the next 20 years, breast cancer cases are expected to soar four times higher in the Middle East. Breast cancer affects Saudi women at an early age compared with developed countries, imposing socio-economic burdens. Most cases are detected at later stages, leading to lower rates of recovery [8]. The awareness of breast cancer plays an important role in early detection and prevention of the disease, and primary healthcare centers are the first level of contact between women and families within the healthcare system. An increase of knowledge about breast cancer screening methods will lead to early intervention and diagnosis of breast cancer as well as increased survival. Our study showed a low-level knowledge of breast cancer in general, mammogram, clinical breast examination, and breast self-examination among female patients attending primary healthcare centers. Our findings can be compared with a previous study in India that showed more than half of women were aware of breast cancer, but less than half of them were unaware of early detection methods [9]. In addition, a study in Abha (Saudi Arabia) demonstrated that less than half and few of the women have heard of breast self-examinations and mammograms, respectively [10]. Another study of female teachers in Buraidah (Saudi Arabia) showed that they had limited knowledge about breast cancer [11]. Also, a few of women were knowledgeable about breast self-examination, clinical breast examination, and mammogram [12]. Therefore, these studies may lead healthcare providers to encourage women to practice breast cancer screening methods to decrease the incidence of breast cancer.

The present study showed that a low percentage of women have not been trained to perform breast self-examinations. In a similar study, only a third of women had performed breast self-examinations [10], whereas in an additional study, more than a third of women performed breast self-examinations [12]. To improve the awareness of breast cancer in women, healthcare providers teach women the correct way to perform breast self-examinations and inform them of the normal structure of the breast to enhance their ability to identify abnormalities and how to report the abnormalities to healthcare providers. Clinical breast examinations are another method for breast screening. The results from our study can be compared with previous studies in Saudi Arabia that found that few of women in Abha [10], less than third in Buraidah and Al Hassa [11, 13], received clinical breast examinations.

Our findings were similar to previous studies that indicated a low percentage of women have had a mammogram [10, 11, 14–16]. The American College of Obstetricians and Gynecologists and the American Cancer Society offer mammograms at the age of 40 years old [16]. In Saudi Arabia, the government provides mammograms free to the population through healthcare providers and other charity organizations, such as the Zahra Breast Cancer Association. Even though the government provides mammograms for free, still the majority of women do not utilize these services.

In the present study, the main source of information about breast cancer was from social media, which was similar to a previous study [10]. These findings have led us to use social media to help increase the knowledge of breast cancer screening methods and to encourage the population to perform these methods. The Ministry of Health in Saudi Arabia hosted an annual event that hoped to raise the awareness of breast cancer during International Breast Cancer Awareness Month that was held in October [8].

## Conclusion

In the present study, we observed that the majority of women in Najran City demonstrated a poor knowledge of breast cancer and breast cancer screening methods. Additional effort should be put forth through women’s healthcare providers, social media, schools, universities, shopping malls, famous people, and hospitals to increase the awareness of breast cancer screening through the importance of primary healthcare for early detection of breast cancer in the early stages.

## Electronic supplementary material

ESM 1 (DOCX 15 kb)
