# Supplementary material for: Knowledge, Attitudes, and Practices of Breast Cancer Screening Methods Among Female Patients in Primary Healthcare Centers in Najran, Saudi Arabia
Source: J Cancer Educ. 2018 Sep 6;34(6):1167–72. doi: 10.1007/s13187-018-1423-8 (PMC6882780; doi:10.1007/s13187-018-1423-8)
Supplement: Supplementary file 1 — (DOCX 15 kb) [file 13187_2018_1423_MOESM1_ESM.docx]

**Online Resource 1. Demographic and medical characteristics of female patients attending Primary Health Care Centers in Najran, Saudi Arabia**

| **Demographic and medical characteristics** | | **No** | **%** |
| --- | --- | --- | --- |
| **Age in years** | <30 | 158 | 31.6% |
|  | 30-39 | 216 | 43.2% |
|  | 40-49 | 100 | 20.0% |
|  | 50+ | 26 | 5.2% |
| **Nationality** | Saudi | 418 | 83.6% |
|  | Non-Saudi | 82 | 16.4% |
| **Education level** | Illiterate | 65 | 13.0% |
|  | Primary | 78 | 15.6% |
|  | Secondary | 154 | 30.8% |
|  | University | 146 | 29.2% |
|  | Postgraduate | 57 | 11.4% |
| **Occupation** | Working | 107 | 21.4% |
|  | Housewife | 393 | 78.6% |
| **Marital status** | Single | 58 | 11.6% |
|  | Married | 351 | 70.2% |
|  | Divorced | 56 | 11.2% |
|  | Widow | 35 | 7.0% |
| **Medical history** | Free | 359 | 71.8% |
|  | Diabetes Mellitus | 42 | 8.4% |
|  | Hypertension | 23 | 4.6% |
|  | Hypothyroidism | 33 | 6.6% |
|  | Cardiovascular disease | 7 | 1.4% |
|  | Other | 36 | 7.2% |
| **History of benign breast disease** | Yes | 35 | 7.0% |
|  | No | 465 | 93.0% |
| **Age of marriage** | < 20 | 180 | 40.7% |
|  | 20-29 | 211 | 47.7% |
|  | 30-39 | 48 | 10.9% |
|  | 40+ | 3 | .7% |
| **Parity** | Nulliparous | 78 | 17.6% |
|  | Less than 4 | 248 | 56.1% |
|  | 4+ | 116 | 26.2% |
| **Age of menarche** | Unknown | 110 | 22.0% |
|  | < 12 years | 158 | 31.6% |
|  | 12-14 | 191 | 38.2% |
|  | After 14 years | 41 | 8.2% |
| **Age of menopause** | Still menstruating | 447 | 89.4% |
|  | Before 50 years | 20 | 4.0% |
|  | After 50 years | 11 | 2.2% |
|  | Don't know | 22 | 4.4% |
